# Rapid and sensitive magnetic field sensor based on photonic crystal fiber with magnetic fluid infiltrated nanoholes

**DOI:** 10.1038/s41598-022-13873-z

**Published:** 2022-06-11

**Authors:** Saeed Azad, Satyendra Kumar Mishra, Ghasem Rezaei, Ricardo Izquierdo, Bora Ung

**Affiliations:** 1 Department of Electrical Engineering, École de technologie supérieure, Montreal, H3C 1K3 Canada; 2 Université Laval, Center for Optics, Photonics and Lasers (COPL), Quebec, G1V 0A6 Canada; 3 Department of Physics, College of Sciences, Yasouj University, Yasouj, 75918-74934 Iran; 4 Department of Electrical Engineering, LACIME, Montreal, H3C 1K3 Canada

**Keywords:** Nanoparticles, Fibre optics and optical communications, Optical sensors

## Abstract

A fast response time (0.1 s) magnetic field sensor has been demonstrated utilizing a photonic crystal fiber with nano-size air holes infiltrated with polyethylene glycol based magnetic fluid. The effect of magnetic nanoparticles concentration in the fluid on the magneto-optical sensor performance and its dependence under varying magnetic-field loads was investigated in detail. In particular, the sensor response was analytically modelled with a Langevin function with a good fit (R$\ge$0.996). A threshold sensing point as low as 20 gauss was recorded and a detection range of 0–350 gauss was demonstrated by means of optical transmission measurements. The experimental results were validated by theory using a waveguide light transmission model fed by finite-element method simulations of the principal guided modes in the infiltrated fiber sensor. The simple interrogation scheme, high sensitivity and quick response time makes the proposed hybrid fiber-optic magneto-fluidic probe a promising platform for novel biochemical sensing applications.

## Introduction

With the advent of the internet of things, wearable sensors and personalized medicine, there is growing demand for compact and reliable sensors to provide biosensing and environmental monitoring to users and artificially intelligent beings. Among various kinds of optical fiber based sensors, specialty fibers infiltrated with magnetic fluid has recently attracted scrutiny towards the development of highly sensitive and compact magnetic field sensors. Magnetic field sensors have been extensively used in electric current measurements, in metallurgy, power industry, in biomedical detection, for the oil and gas exploration as well as aviation industry^1–3^. The most common methods rely on magneto-transistor, magneto-resistive, fluxgate or the Hall effect to detect and measure magnetic fields^4–6^. These sensors exhibit some drawbacks related to their power consumption, limited multiplexing, cost, miniaturization and remote monitoring capabilities. In addition, surrounding electrical field sources are prone to introduce noise via electromagnetic interference to the electronic circuits^7^.

Compared to conventional sensors, optical fiber based magnetic field sensors offer promising key benefits such as a compact size, immunity to electromagnetic interference, remote monitoring and multiplexing capabilities through optical networks modalities, high reliability and sensitivity. Initial fiber-optic magnetic field sensors reported in the last four decades employed magnetostrictive materials in conjunction with Mach–Zehnder interferometry while other schemes exploit changes on the state of polarization of the light^8–11^.

Meanwhile with the growth of nanotechnology and the advent of liquids functionalized with nanoparticles, emerging applications of so called magnetic fluids (MF) are studied in the sensing field. A MF is a liquid typically composed of single-domain magnetic nanoparticles (MNPs) coated with surfactant in suspension within a liquid carrier, and with engineered physicochemical properties including magnetic susceptibility, polydispersity, and dipolar interactions. Owing to its customizable magneto-optical properties, MFs have been applied in a variety of photonic devices, including optical gratings^12^, optical switches^13^, modulators^14^, couplers^15^ and magnetic field sensors^16^.

The ability to exhibit a magnetic-field-dependent refractive index (RI)^17,18^ that is attributed to the microstructural distribution of MNPs inside the MF-is a key parameter used in many sensing applications. Accordingly, different configurations of optical fibers in conjunction with MF have been well studied as magnetic field sensors. They can be used in three different configurations, initially in the form of the MF thin film at the end facet of optical fiber cross section, as the cladding of an etched/tapered fiber (in the middle section) and finally as the filling material inside the fiber. For the first configuration, several Fabry–Pérot based sensors that incorporate MF within a section of optical fiber were reported^19,20^. This technique suffers from sensitivity to thermal expansion and a complicated process to calculate and fabricate the correct cavity dimensions. Those problems were solved in etched tapered fibers^21,22^, however these thinned fibers are very fragile owing to their low mechanical strength. Finally, by injecting the MF inside the fiber, the original microstructured features are not only preserved but the whole fiber also offers an extended interaction area that enhances the sensitivity^23^. In this work, we present a special photonic crystal fiber (PCF) with nanometer-scale air holes infiltrated with MF. Sensor performances including sensitivity, threshold and saturation points, response/recovery times were studied in detail for various concentrations of the MF. This work is organized as follow: section “Fabrication process and operation principle” describes the PCF infiltration process and sensing principle. In section “Results and discussion” the experimental investigation on the effect of MF concentrations in sensor responses are considered. Also, numerical simulations regarding output power, which is then followed by a comparison between the experimental and simulation results were done. The proposed functional sensor with features such as compact size and fast response/recovery time may find applications in future biochemical and industrial sensing.

## Fabrication process and operation principle

**Figure 1:**
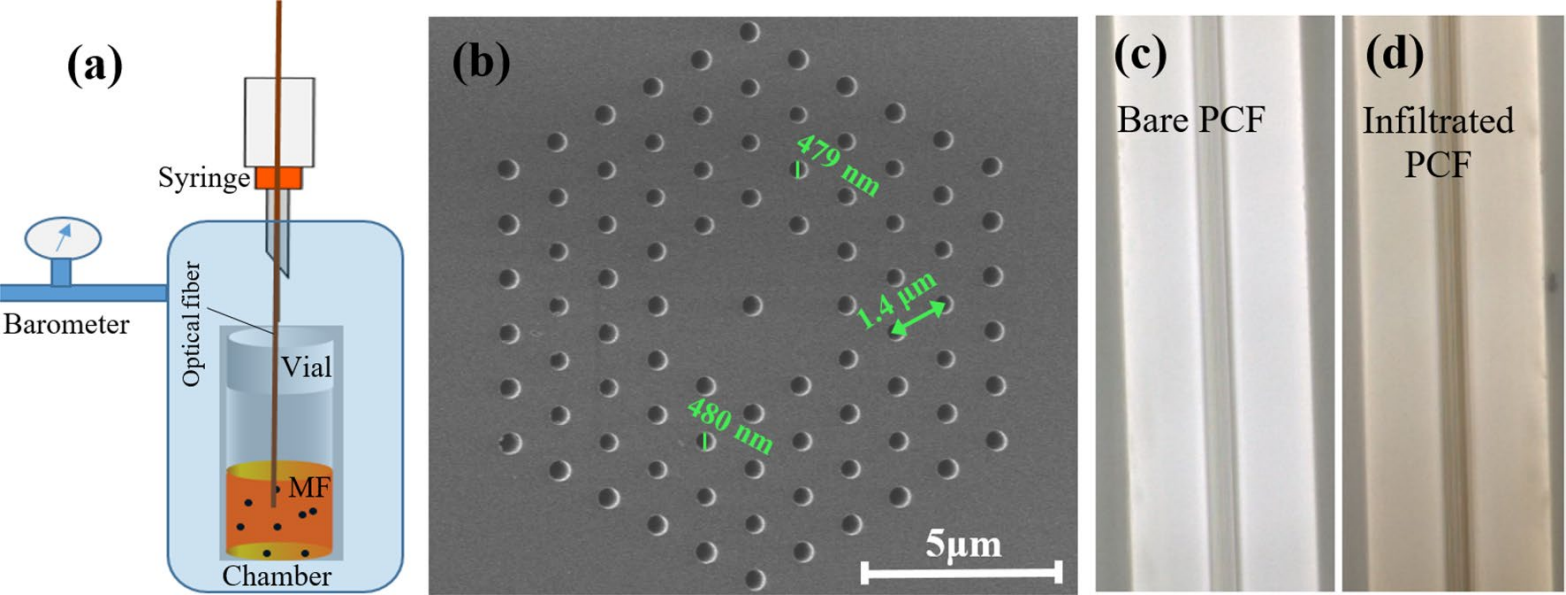
(**a**) Schematic of experimental setup for filling the PCF, (**b**) Cross section SEM image of PCF, (**c**) Optical microscope image of Bare PCF and (**d**) infiltrated side views of the PCF.

Figure 1a,b depict the schematic experimental setup for filling the PCF with the MF (ferromagnetic MNP of 10 nm average size in polyethylene glycol solution from Ferrotec, USA). The flat-cleaved end of a 15 cm PCF was immersed perpendicularly in 2 ml sample vial containing the MF. The MF was successfully infiltrated into the air holes and throughout the whole length of the PCF based on Poiseuille law^24^ under which the induced pressure gradient between the two ends of the fiber results in a laminar flow of MF [Fig. 1c,d].

Figure 1b shows the SEM cross-section of the used PCF which features a holey cladding made of air holes arranged in a hexagonal lattice pattern with 1.4 mm pitch and average hole diameter of 480 nm . In order to apply a uniform magnetic field on the side of the MF-infiltrated PCF, a plate shaped magnet (KJ Magnetics, USA) was placed next to the sensing region at precisely defined distances from the fiber. A Hall probe-based magnetometer (KOSHAVA 5 model, Wuntronic GmbH) was employed to monitor the magnetic field strength and calibrate the magnetic field fiber-optic sensor. The output beam profile as well as transmitted power at the output of optical fiber were monitored via a CCD camera and optical power meter respectively.

**Figure 2:**
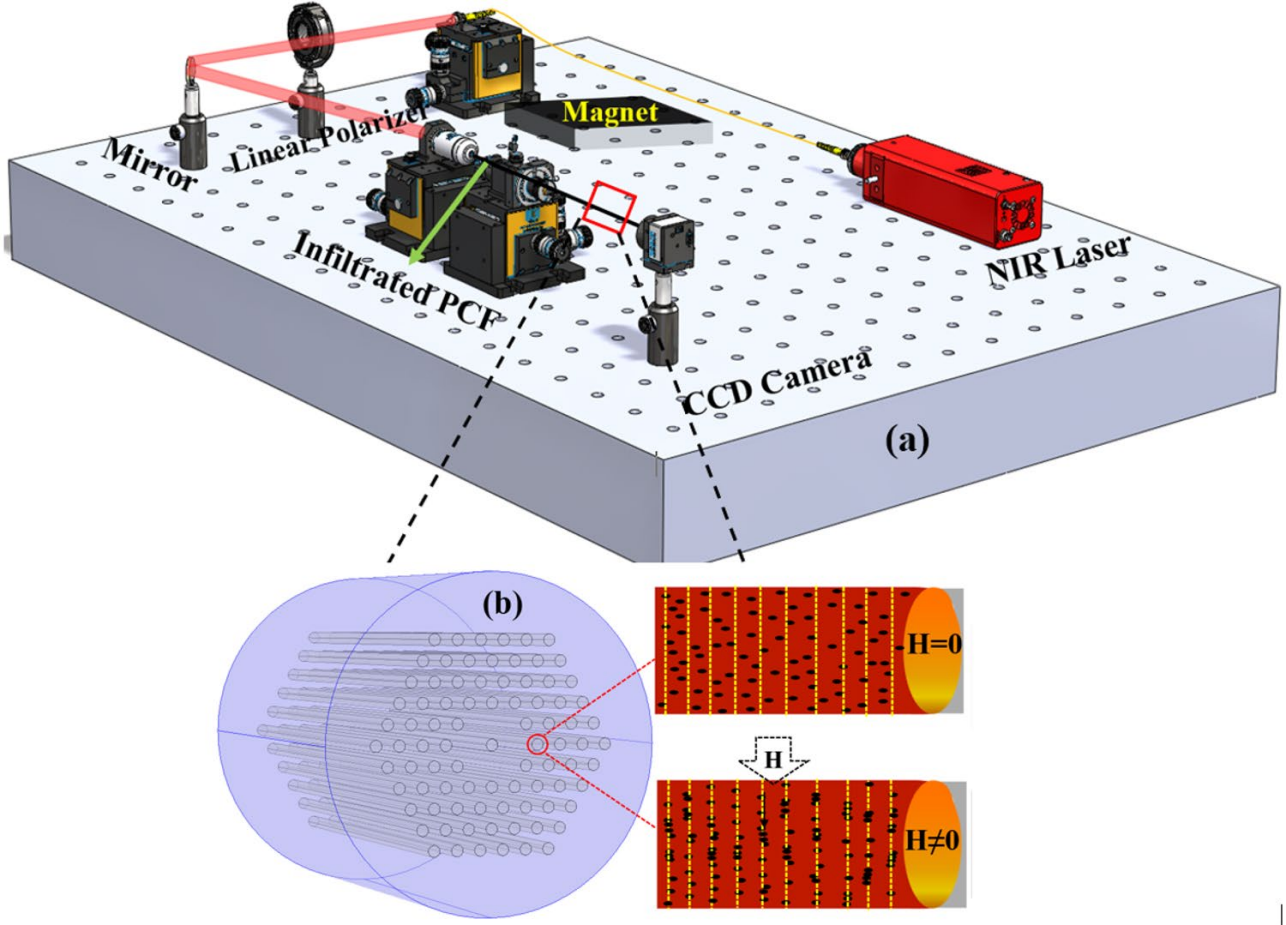
Schematic illustration of (**a**) experimental setup, (**b**) magnetic nanoparticles arrangement within the holes of the PCF with (bottom image) and without (top image) the application of an external magnetic field.

As shown in Fig. 2b, when the infiltrated PCF was exposed to the magnetic field the spatial distribution of MF transforms from randomly homogeneous to an ordered field dependent pattern. The MNPs tend to agglomerate and form chain-like clusters along the direction of magnetic field^25^ owing to Néel and Brownian relaxation. This phenomenon induces a refractive index (RI) change of the MF that depends on the exerted magnetic field strength^26,27^.

## Results and discussion

It is well known that MFs possesses high optical absorption in the visible spectrum^28^ as well as a high absorption band at 1470 nm wavelength. The absorption band is related to the orbital transition process in magnetite particles^29^. Therefore, to benefit from the RI tunability of MF in sensing applications, the geometry of the optical waveguide plays an important role. For example, in the case of a PCF with large air holes, a larger fraction of optical power would be absorbed resulting in very high optical loss. In this work, the use of a special PCF with very small air holes enabled light transmission in the range of 800–1000 nm. The experimental testing setup is depicted in Fig. 2a.

The incident light from a near infrared laser source ($\lambda$= 976 nm, Thorlabs, Pigtailed Butterfly Package) was coupled into the PCF through a combination of objective lenses. The transmitted light power and output beam profiles were recorded via an optical power meter and CCD camera, respectively. A linear polarizer was tuned so as to optimize the interaction of light with the MF: when the direction of E-field is parallel to the direction of exerted magnetic field (H) the induced change in optical absorption is almost twice that of the case when the E-field is perpendicular to the direction of applied H-field^29^.

### Influence of MF concentration

**Figure 3:**
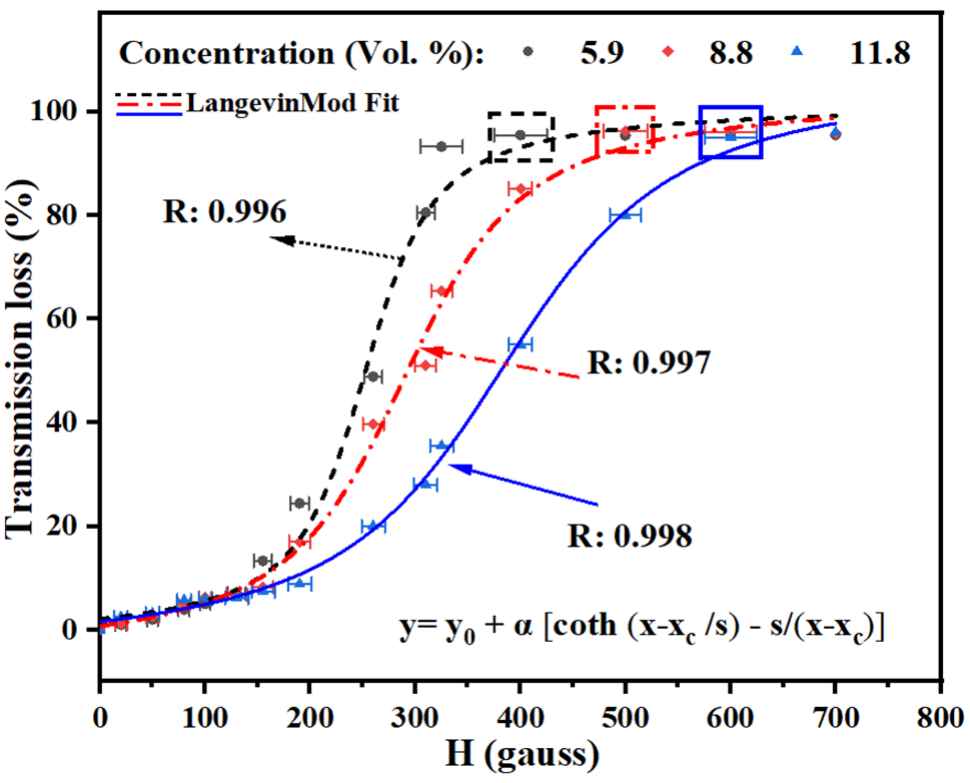
Transmission loss versus magnetic field for samples with various Vol.% concentration of MNPs.

The proposed sensor operates based on RI variation and this phenomenon strongly depends on the volume fraction of magnetite particles and the liquid carrier (water, organic solvent, etc.). In this regard, the used polyethylene glycol-based MF with superparamagnetic properties offers higher colloidal stability compared to water based MFs. In this experiment, three different concentrations of MF with 5.9, 8.8 and 11.8 Vol.% of magnetic particles were considered. As shown in Fig.  3 the transmitted optical power through the infiltrated PCF exhibits a strong dependence with the applied magnetic field strength. The sensor saturation point (identified by square markers in Fig.  3) increased with the concentration of MNPs, which is attributed to the saturation magnetization $M_{s}$ of the MF that follows a linear dependence with the concentration. Generally, the magnetization of superparamagnetic materials is described by a Langevin function under magnetic field^30^.

Correspondingly, the experimental results in Fig. 3 were fitted with a Langevin function with a good degree of confidence ($R \ge 0.996$). The proposed sensor exhibited a limit of detection$\le$16 gauss within the effective sensing range (i.e. below saturation point).This demonstration of the magnetic-field modulation of transmitted light via the superparamagnetic response of magnetite NPs^31^ points to the potential of using the proposed MF-infiltrated PCF waveguide in magneto-optical sensing applications.

**Figure 4:**
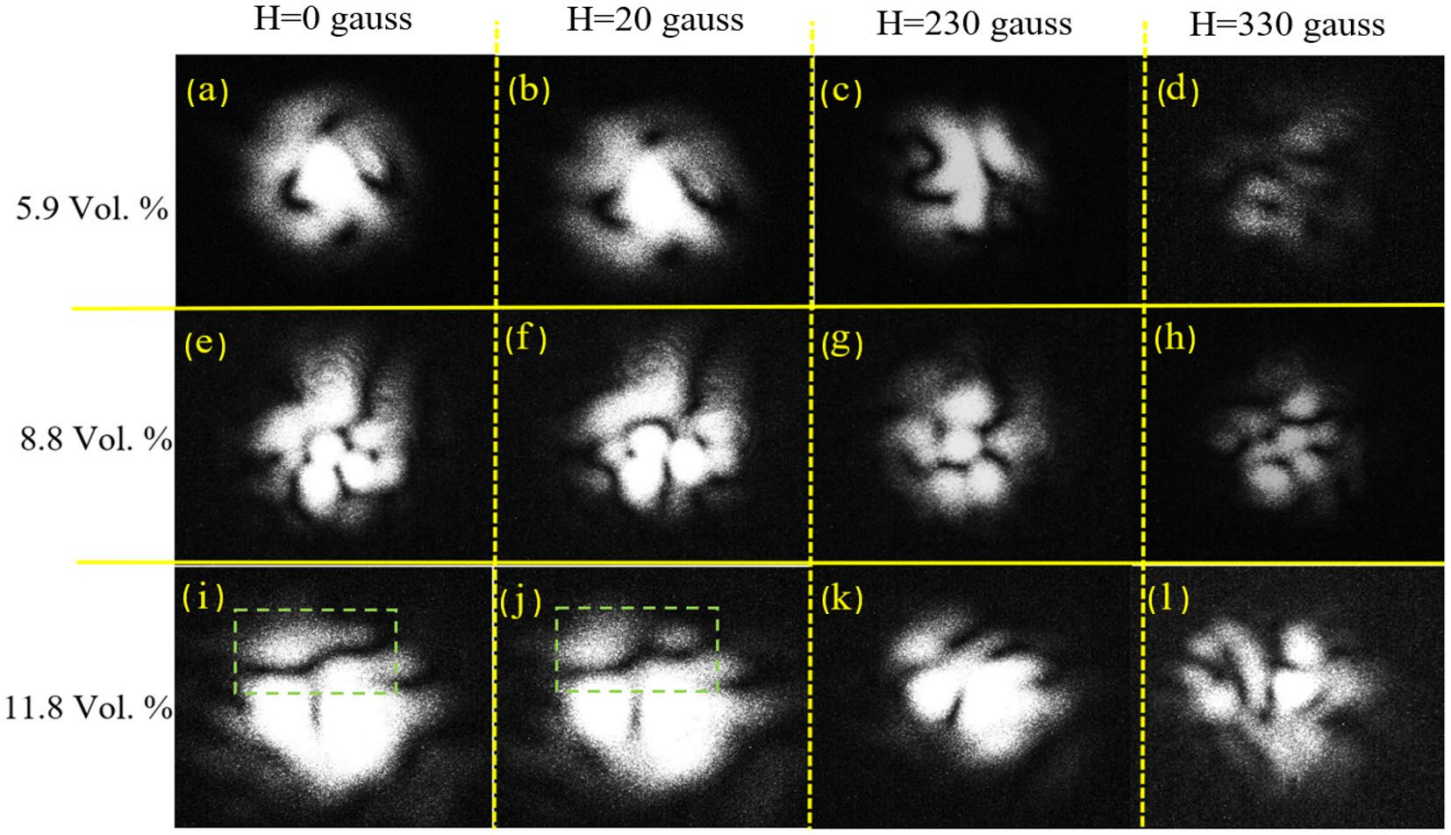
Output beam pattern intensity related to the PCFs infiltrated with: (**a**–**d**) 5.9 Vol.%, (**e**–**h**) 8.8 Vol.% and (**i**–**l**) 11.8 Vol.% concentrations of MNPs under applied magnetic fields of 0, 20, 230 and 330 gauss.

Figure  4 presents the CCD camera images related to the output beam pattern intensity of the infiltrated PCF with various concentrations of MF and submitted under varying magnetic field strengths. This figure provides a clear visual support to the fact depicted in Fig. 3 that transmission losses increase with the applied magnetic field for all three MF concentrations investigated. In the absence of an external magnetic field, individual magnetite particle can be described as a single domain magnetic dipole with permanent moment. While in the presence of low magnetic field (20 gauss), the sensor containing the highest concentration of MNPs showed distinctive pattern changes highlighted by green dashed rectangles in Fig. 4i,j. An explanation is that at higher volume percentages of MNPs, because the free distance between the centers of two dipoles is lower the portion of attractive polar energy is larger than the thermal energy such that dipole-dipole interactions dominate^32^. The latter phenomenon results in small RI changes, which in turn leads to the observed variation in the output beam pattern.

### Dynamic response of the sensor

**Figure 5:**
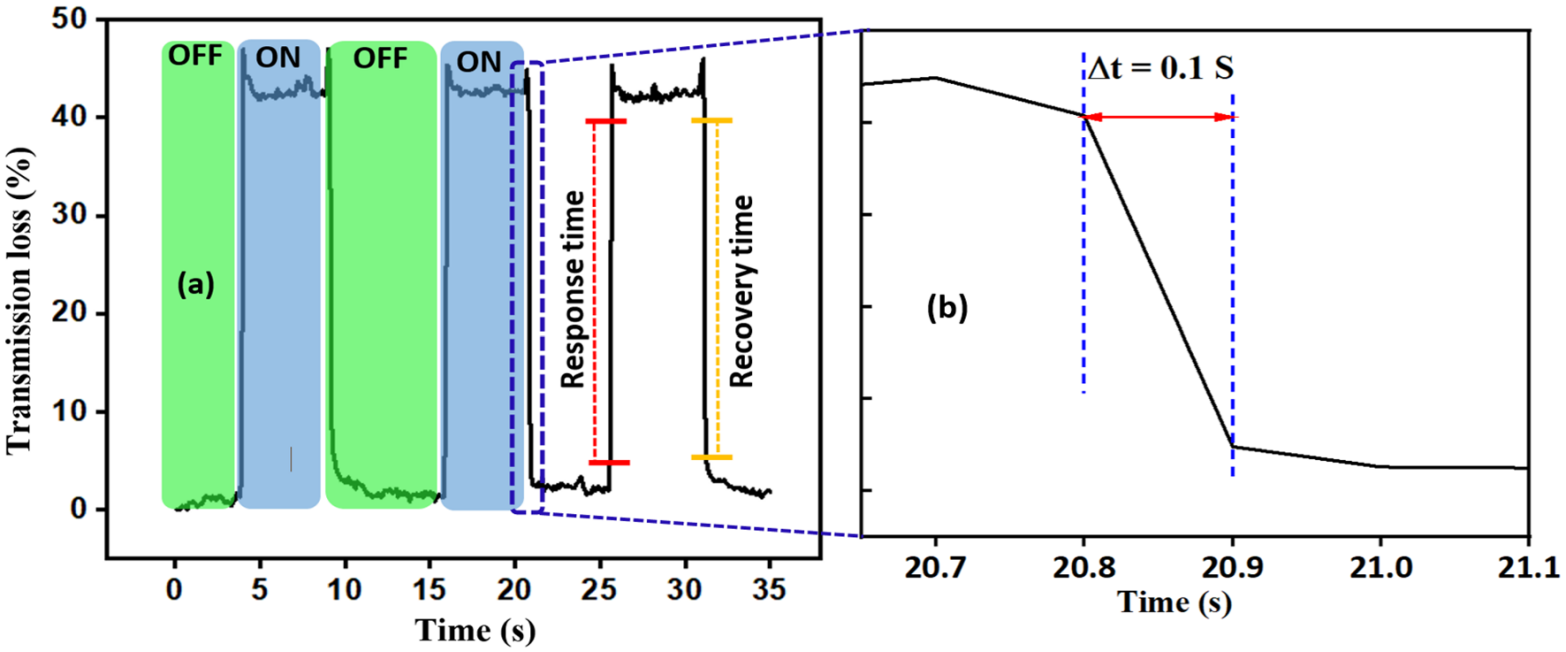
(**a**) Dynamic response of the infiltrated PCF with 5.9 Vol. % MF in H=250±8.7 gauss, (**b**) Close-up view with finer resolution of the response time region.

The reaction time in sensor applications is an important parameter. In order to evaluate the dynamic response of our sensor, equal length of infiltrated PCFs with different concentrations of MF were exposed to constant magnetic field of 250±8.7 gauss.To ensure the stability and repeatability of sensor responses, the samples were exposed to magnetic field consecutively for three repetitions. The samples containing the lowest concentration of magnetic particles (5.9 Vol%) exhibited very fast response time (time interval during which the transmitted optical power changes from 90 to 10% of its variation) of 0.1s and recovery time (in reverse of response time definition) of also 0.1s as shown in Fig. 5.

Sample with 8.8 Vol. % showed 0.16s response/recovery time. Although MF with the highest concentration (11.8 Vol.%) showed a longer response time of 150s and recovery time of 9s . The latter observed long response time is explained by the growing short-range repulsive forces that increase at high concentration levels of MNPs and which, in turn, slow down the attractive dipole–dipole interactions responsible for the formation of chain-like clusters [depicted in Fig. 2b]. It should be noted that the response and recovery times were not affected by the strength of the applied magnetic field. In Table 1 we summarized the performance of the main sensing specifications reported in the recent literature in comparison to the present work. The compiled results show that the proposed PCF sensor compares favorably in terms of sensitivity and response time.

**Table 1:** Critical parameters in sensing performance based on optical fiber in conjunction with MF for sensing magnetic field.

Detection mechanism	Optical fiber configuration	Ferrofluid	Detecting range	Response time	Working wavelength (nm)	Ref.
Wavelength shift	eFBG$^{a}$	Synthesized MF based on ${{\mathrm{Fe}}_3 {\mathrm{O}}_4}$	0–25 mT	15 s	Not mentioned	^34^
Cladding mode intensity	FBG$^{b}$/ TCF$^{c}$	Ferromagnetic Particles	7–15 mT	30 s	1539–1546	^35^
Wavelength shift	Tapered PCF$^{d}$	EMG 507	10–60 mT	30 min	1550	^21^
Evanescent waves	POF$^{e}$	Synthesized MF composite	2.5–150 mT	0.7s	550	^22^
Transmitted power variation	P MZI$^{f}$	EMG 605	0–40 mT	Not mentioned	1550	^36^
Optical loss	PCF$^{g}$	PBG 300	0–35 mT	0.1 s	976	Present study
Wavelength shift	SNS$^{h}$	EMG 605	4–10 mT	Not mentioned	1530–1560	^37^

^a^Etched fiber Bragg grating.

^b^Uniform fiber Bragg grating.

^c^Thin core fiber.

^d^Photonic crystal fiber.

^e^Polymer optical fiber.

^f^Mach–Zehnder interferometer.

^g^Photonic crystal fiber.

^h^Single mode-no-core-single mode.

### Sensor output modelling

We also modeled the principal waveguiding mechanism of the infiltrated PCF. Due to the small RI contrast between the silica glass fiber structure and the MF, we expect leakage of the guided light into the holey cladding region, as evidenced by the recorded output intensity patterns in Fig. 4.

In order to model this peculiar waveguiding, finite-element method (FEM) simulations using COMSOL Multiphysics were performed. A uniform 480 nm diameter of PCF holes was assumed along with a pitch value of 1.4 $\upmu {\mathrm{m}}$ inside the 125 $\upmu {\mathrm{m}}$ diameter silica PCF coated with acrylate protective jacket (250 $\upmu {\mathrm{m}}$ diameter). Moreover, the refractive index of different MFs was measured via a digital refractometer (Kruss DR301-95) at 589 nm wavelength. It was observed that the RI increased linearly with increasing concentration of samples. That is for 5.9, 8.8 and 11.7 Vol. % MF the measured RI were 1.4276, 1.4707 and 1.493, respectively.

The latter values of RI were used in the simulations since changes in RI of the MF at 589 nm compared to a wavelength of 976 nm are negligibly small as well as exhibit a similar trend with respect to changes in MF concentration. The first five principal guided modes were selected for each value of applied magnetic field, and the corresponding $n_{eff}$, loss ($\alpha$), E-field as well as H-field components were calculated with the FEM mode solver.

Subsequently, the transverse E-field distribution at the output facet of the fiber sensor of length *L* was modeled as the coherent superposition of the *N* guided modes as described by the following equation :

1 $$\begin{aligned} E_{output} (x,y,\omega ) =\sum _{j=1}^{j=N} C_j.E_j (x,y,\omega )e^{(\frac{i\omega }{c})(n_eff,j) L} e^{\frac{-\alpha _j L}{2}} \end{aligned}$$

where $E_j = (E_j^x,E_j^y)$ are the *x* and *y* transverse field components, while $n_{eff,j}$ and $\alpha _j$ denote the real effective index and the power loss coefficient of the $j-th$ guided mode at a given frequency $\omega$. The variable $C_j$ stands for the normalized amplitude coupling coefficients calculated from the overlap integral of the input Gaussian beam and overlap integral of the respective modal distributions of the $j-th$ mode :

2 $$\begin{aligned} C_{j}=\frac{1}{4}\int \left[ E^{x^{*}}(x,y)_{input}.H_j^y (x,y)+ E_j^x (x,y).H^{y^{*}}_{input}(x,y)\right] dxdy \end{aligned}$$

where the modal fields were properly normalized to unit power through via $\frac{F}{\sqrt{\frac{1}{2}\int Rel{(E_t\times H_t^*)} dxdy}}$, where *F* stands for the (E or H) field component of the electromagnetic field vector. The used 976 nm laser is linearly polarized such that an x-polarized Gaussian beam of radius $\sigma =7\upmu m$ was considered as the input source with optical power P: in the simulations:

3 $$\begin{aligned} E_{input}(x,y)\approx {\hat{x}}.\sqrt{\frac{2P}{\pi \sigma ^2 n_{clad}}}exp\left[ \frac{-(x^2+y^2)}{2\sigma ^2}\right] \end{aligned}$$

4 $$\begin{aligned} H_{input}(x,y)\approx {\hat{y}}.\sqrt{\frac{2P n_{clad}}{\pi \sigma ^2 }} exp\left[ \frac{-(x^2+y^2)}{2\sigma ^2}\right] \end{aligned}$$

**Figure 6:**
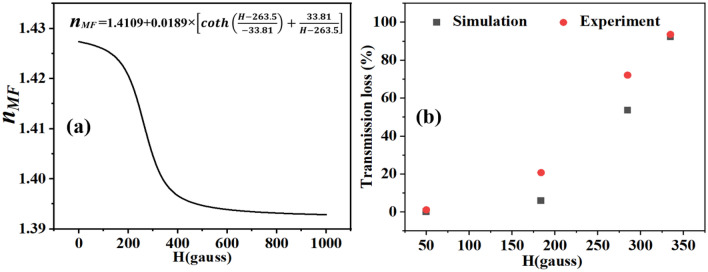
(**a**) Modeled $n_{MF}$ versus applied magnetic field, (**b**) Comparison between simulation and experimental data regarding the optical transmission loss in the MF-infiltrated PCF as a function of applied magnetic field.

Therefore by using Eq. (1) an expression can be derived for the transmitted power in the infiltrated PCF sensor:

5 $$\begin{aligned} T_{sensor}(\omega )\approx \left| E_{output} (x_0,y_0,\omega ) \right| =\left| \sum _{j=1}^{j=N} C_j .E_j (x_0,y_0,\omega )e^{\frac{i\omega n_{eff,j}L}{c}} e^{\dfrac{-\alpha _j L}{2}} \right| \end{aligned}$$

where $(x_0,y_0)$ denotes the coordinates of the PCF cross-section center. In order to model the transmitted power in Eq. (5) of our fiber sensor, we used the FEM-simulated first five dominant guided modes that carry$\ge$ 90$\%$ of the transmitted power. Another key simulation parameter that was considered relates to the changes in the refractive index of the magnetic fluid ($n_{MF}$) to implement in our simulations when the applied magnetic field is $H>0$. This relationship between $n_{MF}$ and *H* is a priori unknown. But knowing that the value of $n_{MF}$ obeys a decreasing Langevin function behavior^30,33^and the fact that we measured the value of $n_{MF}$ at *H=0* gauss, we performed a series of FEM simulations that allowed us to find the best fit between the simulated and experimental sensor output transmission loss in Fig. 6b using the model for $n_{MF}$ in Eq. (6) and shown in Fig.  6a. We note that the model in Eq. (6) was derived for a MF concentration of 5.9 Vol.% for which we observed the highest sensor performance. Consequently, all simulations were performed for this specific concentration.

6 $$\begin{aligned} n_{MF}=1.4109+0.0189\times \left[ coth\left( \frac{H-263.5}{-33.81} \right) +\frac{33.81}{H-263.5} \right] \end{aligned}$$

The discrepancies between the experiment and simulations are owed to additional optical scattering within the PCF that are not accounted for in the simulations which assumed a perfectly smooth PCF structure.

## Conclusion

The exquisite precision of mature optical fiber technology in combination with functional fluids tailored with fine magnetic particles makes a hybrid fiber-optic magneto-fluidic probe design desirable for emerging biochemical and environmental sensing applications. In this work, we propose and demonstrate a new type of magnetic field fiber-optic sensor based on a special type of photonic crystal fiber (PCF) with very small submicron-sized air holes infiltrated with a functional magnetic fluid (MF). The resulting fiber-optic probe enabled us to demonstrate a highly sensitive (0–350 gauss), fast (0.1 s response time) and compact magnetic field sensor that can be driven using cost-effective near-infrared laser diodes. The experimental results were well fitted using a Langevin function and explained by a magnetic-field and mode-dependent optical transmission model that was validated by finite-element method simulations. This demonstration provides another step towards novel hybrid magneto-fluidic fiber-optic sensing approaches for biochemical and environmental sensing applications.

## Data Availability

The datasets used and/or analysed during the current study available from the corresponding author on reasonable request.
